# Supplementary material for: Application and measurement of tissue-scale tension in avian epithelia in vivo to study multiscale mechanics and inter-germ layer coupling
Source: Development. 2025 Aug 29;152(16):dev204561. doi: 10.1242/dev.204561 (PMC12448309; doi:10.1242/dev.204561)
Supplement: Supplementary information [file develop-152-204561-s1.pdf]

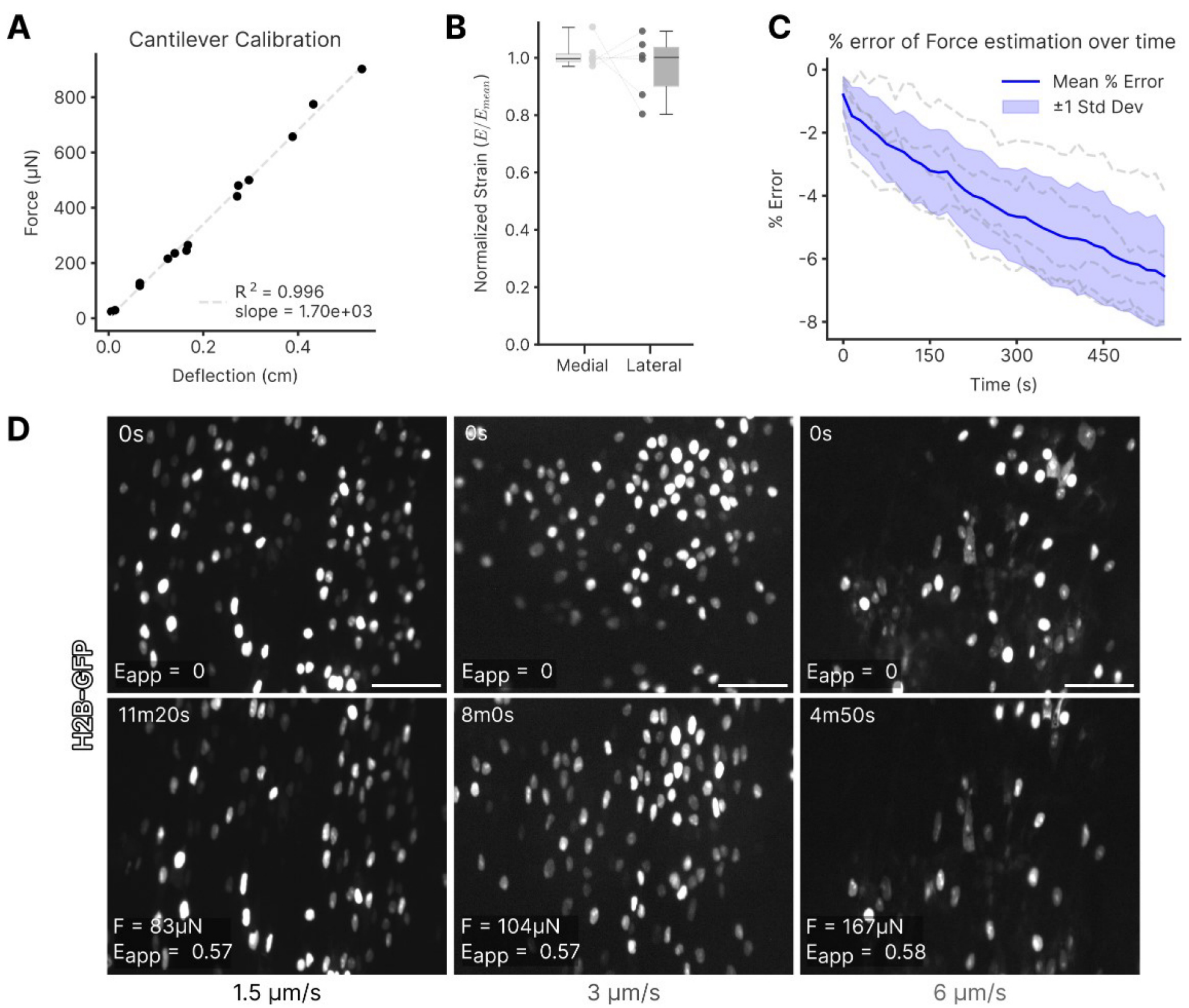

Fig. S1. Beam calibration and strain heterogeneity. (A) Plot of cantilever deflection in function of applied force. **(B)** Paired comparison of mean normalized strains between medial (gut forming) and lateral endoderm in 6 different embryos. **(C)** Percentage of error in force estimation over time. **(D)** Stills from a time lapse of H2B-GFP expressing endoderm cells as applied strain E_{app} is increased at different speed rates (left : l.5µm/s ; middle : 3 µm/s ; right 6µm/s); Scale = 100µm.

Development • Supplementary information

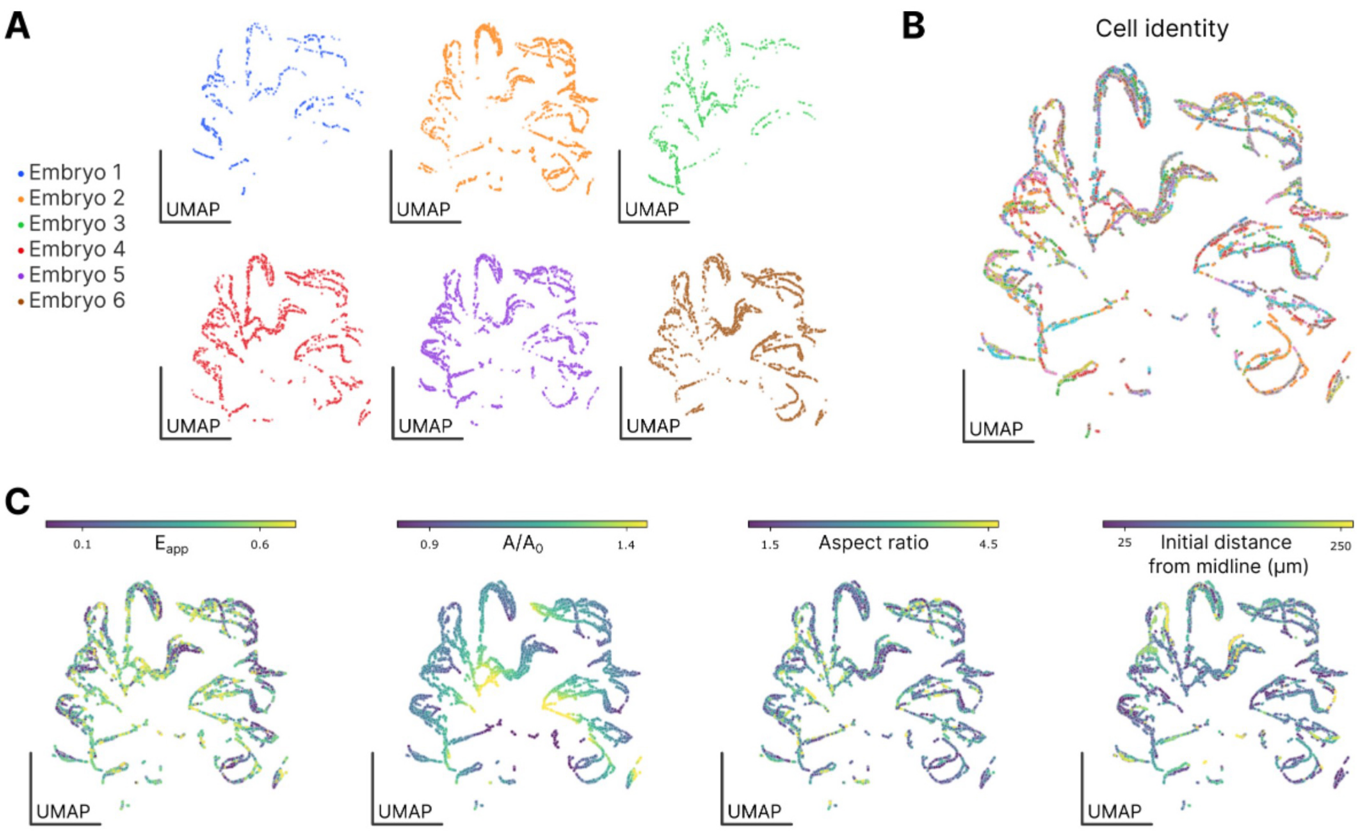

Fig. S2. Distribution of cells in UMAP space. (A) Partitioning of cells from 6 independent experiments in UMAP space reveals each embryo distributed through the UMAP space, indicating that heterogeneity is not likely explained by embryo-to-embryo variability or batch effects. **(B)** Visualization of cell trajectories in UMAP space by random color assignment to individual cells throughout the course of applied deformation. **(C)** UMAP color-coded according to strain , cell normalized area, aspect ratio and initial distance from midline.

Development • Supplementary information

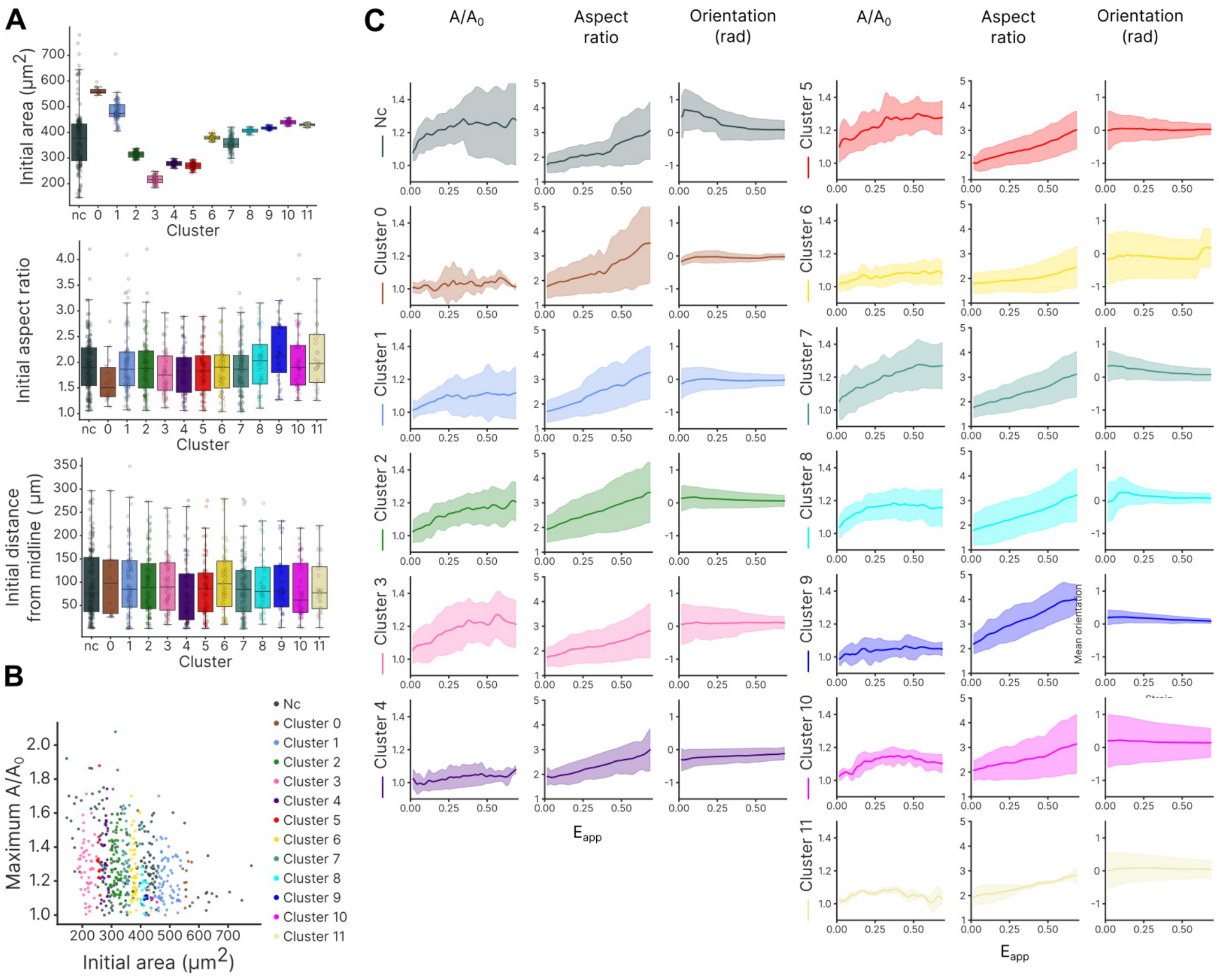

Fig. S3. Evolution of morphometrics by mechanotype. (A) Initial features (area, aspect ratio and distance from midline) observed for the different clusters. 'Ne' correspond to cells that don't belong to any cluster. **(B)** Maximum normalized area reached as a function of initial cell area. Colors denote correspond ing cluster for each cell. **(C)** Normalized area, aspect ratio and orientation as a function of applied strain for each cluster. Solid lines indicate mean and shaded area indicate standard deviation.

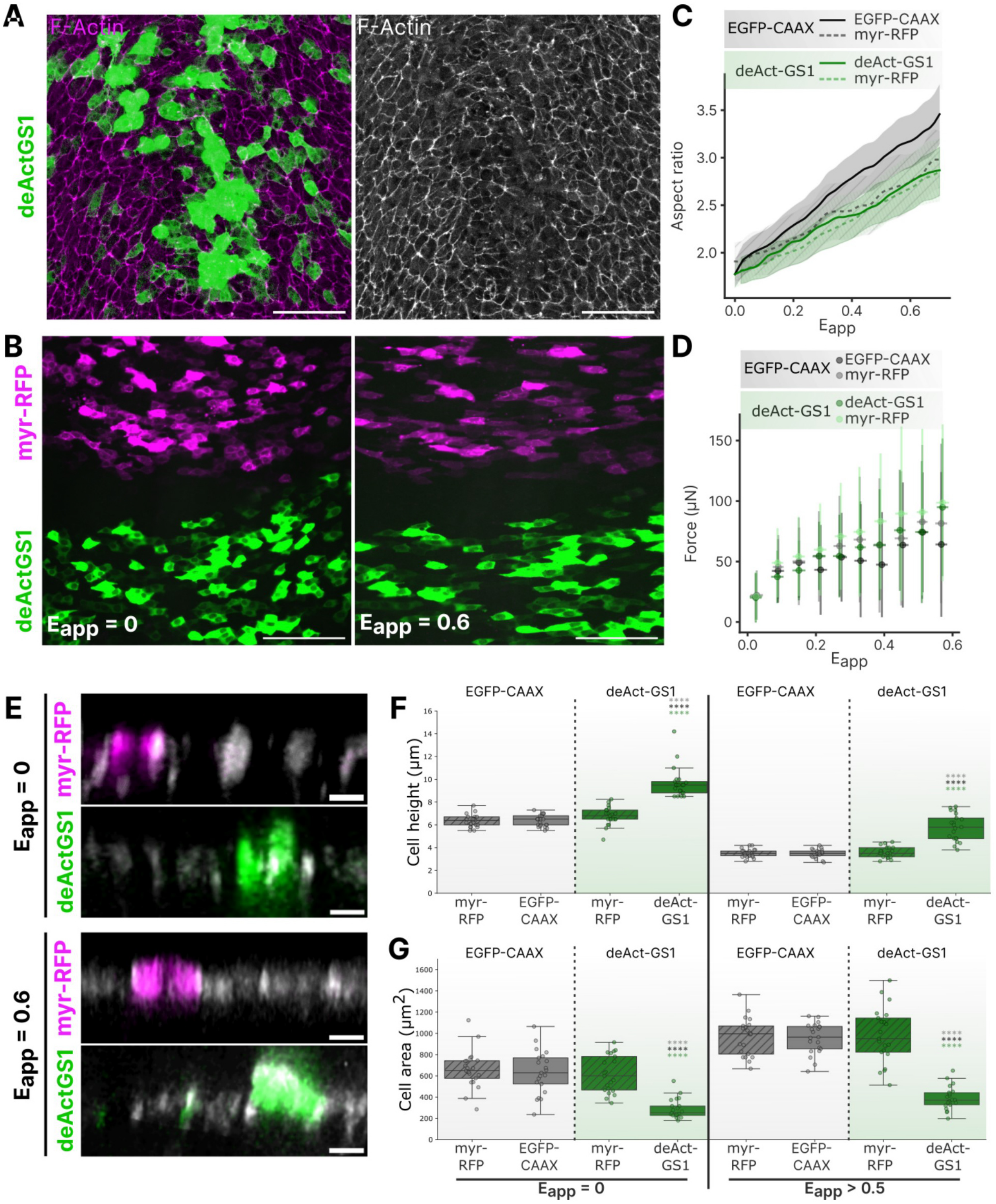
A
F-Actin
F-Actin
deActGS1
C
EGFP-CAAX
EGFP-CAAX
myr-RFP
deAct-GS1
deAct-GS1
myr-RFP
Aspect ratio
Eapp
B
myr-RFP
deActGS1
Eapp = 0
Eapp = 0.6
D
EGFP-CAAX
EGFP-CAAX
myr-RFP
deAct-GS1
deAct-GS1
myr-RFP
Force (μN)
Eapp
E
Eapp = 0
myr-RFP
deActGS1
Eapp = 0.6
myr-RFP
deActGS1
F
EGFP-CAAX
deAct-GS1
EGFP-CAAX
deAct-GS1
Cell height (μm)
myr-RFP
EGFP-CAAX
myr-RFP
deAct-GS1
G
EGFP-CAAX
deAct-GS1
EGFP-CAAX
deAct-GS1
Cell area (μm2)
myr-RFP
EGFP-CAAX
myr-RFP
deAct-GS1
Eapp = 0
Eapp > 0.5

Fig. S4. Disruption of actin through deAct-GSl electroporation. (A) F-Actin staining of H H13 embryo electroporated with deAct-GSl; Scale = 50µm. **(B)** Representative images of neighboring regions of deAct-GSl (green) and myr-RFP (magenta) electroporated cel ls before stretch i ng (left) and at 0.6 applied strain (right); Scale = 100μm. **(C)** Evolution of mean cell aspect ratio with increasing applied strain E_{app} in EG FP-CAAX, deAct-GSl and respective neighboring cells. **(D)** Applied force quantified as a function of tissue strain for control embryos electroporated with EGFP-CAAX and myr-RFP compared to embryos electroporated with deAct-GSl and myr-RFP. **(E)** Representative tra nsverse views of neighboring regions of deAct-GSl (green) and myr-RFP (magenta) before stretching (top) and at E_{nnn} = 0 6 (bottom). Scale = 5nm. **(F-G)** Quantification of cell hei 11:hts **(F)** and cell areas **(G)** at E_{nnn} = 0 (left) and E_{nnn} > 0.5 (right) in EGFP-CAAX, deAct-GS1 and respective neighboring wild type cells (myrRFP). One-way ANOVA followed by TukeyHSD; *****: p<0.0001.

Development • Supplementary information

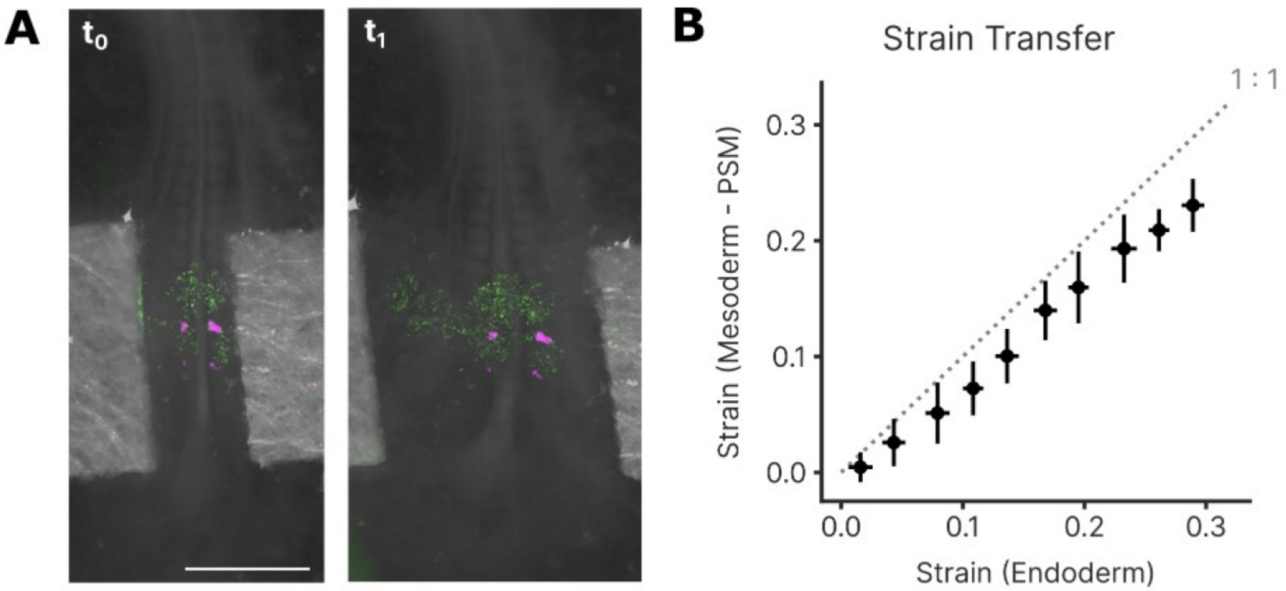

Fig. S5. Strain transfer between endoderm and mesoderm is also observed at the PSM level. (A) Labelling strategy for tracking both endoderm (H2B-GFP electroporations, shown in green) and PSM (Dil injections, shown in magenta) deformations, showing pictures at the start (left) and at the end (right) of a stretching experiment. Scale = 1mm. **(B)** Strain transfer plot between endoderm and mesoderm at the PSM level.

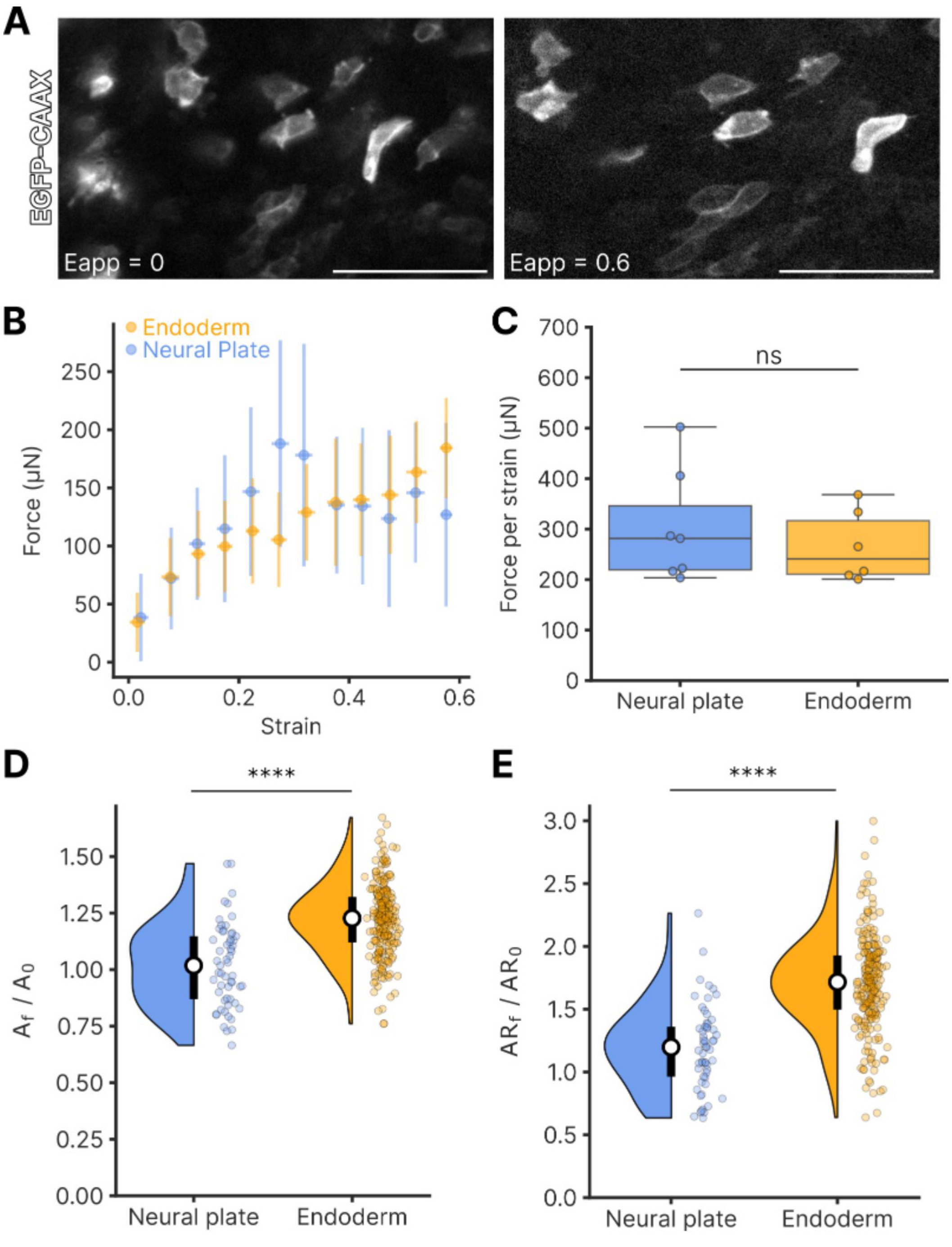

Fig. S6. Application of exogenous tension to the posterior neural plate. (A) Representative images of EGFP-CAAX electroporated neural plate cells before and after application of E_{app} = 0.6 strain. Scale = *100µm.* (B) Applied force quantified as a function of tissue strain for endoderm (orange) and neural plate (blue). (C) Relative stiffness quantified from force-strain curves in (B) (Mann-Whitney U test; ns : p = 0.41). (D) Cell area increase *(At/ Ao)* at E_{app} = 0.6 (Student t-test ; **** : p<0.0001). (E) Cell aspect ratio increase *(ARJ/ ARo)* at E_{app} = 0.6 (Student t-test ; ****: p<0.0001). Endoderm data reproduced from Fig. 2 for comparison.

Development • Supplementary information

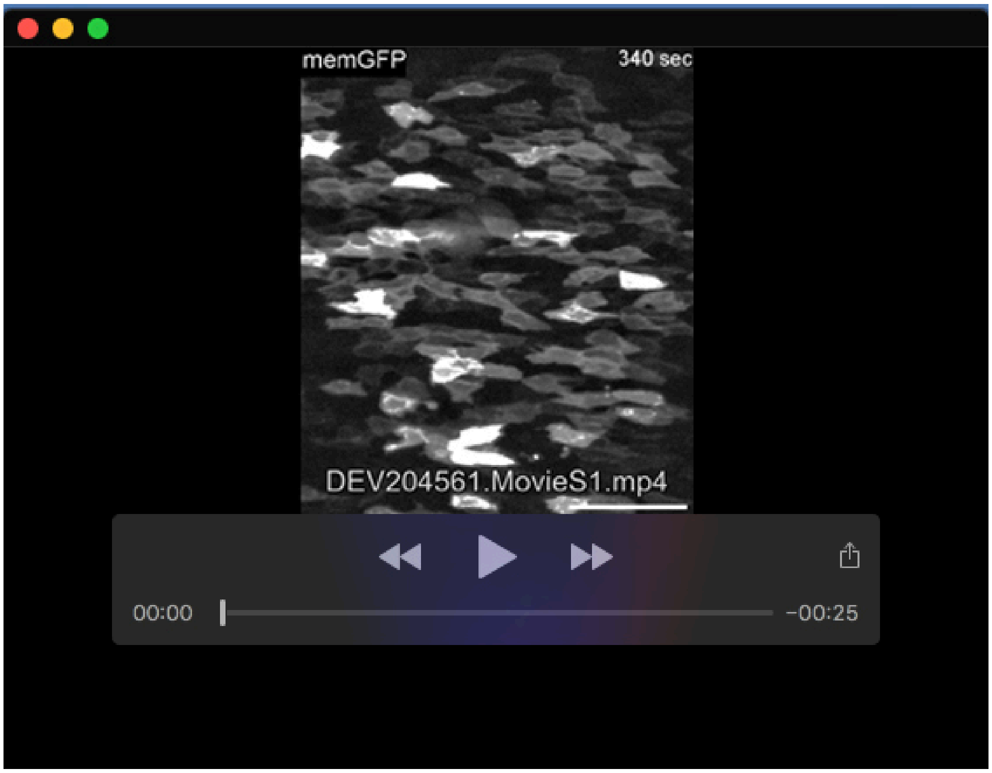

Movie 1. Time-lapse movie of EGFP-CAAX-expressing endoderm cells under increasing applied strain.

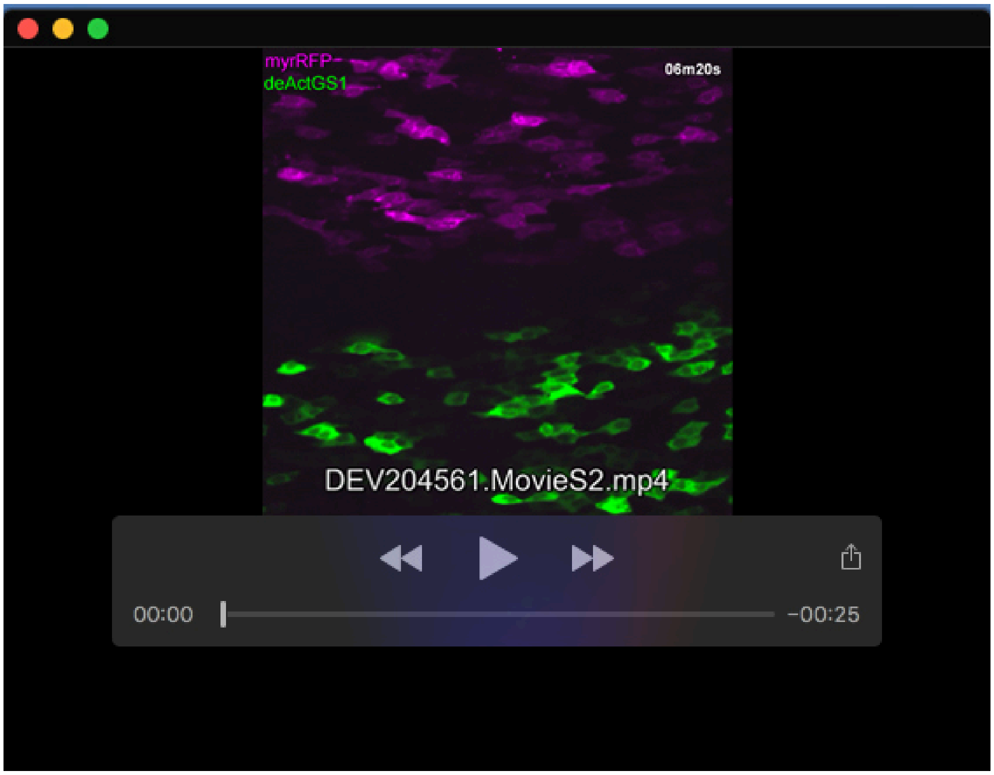

Movie 2. Time-lapse movie of neighboring regions of deAct-GS1 (green) and myr-RFP (magenta) electroporated cells under increasing applied strain.

Development • Supplementary information

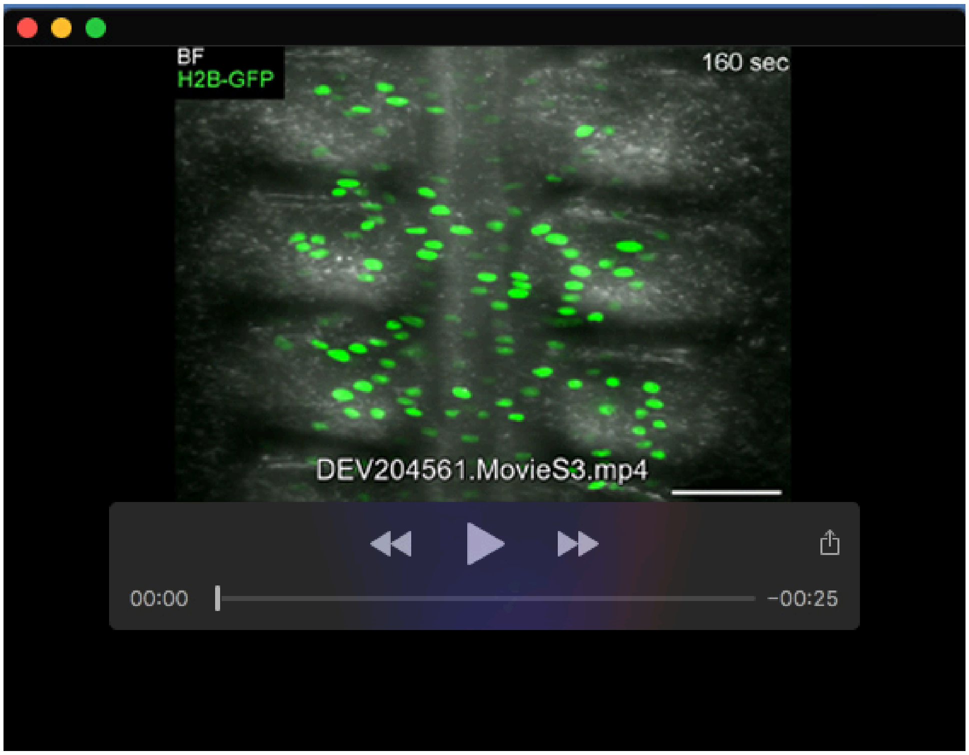

Movie 3. Time-lapse movie of observed strain transfer between endoderm (green) and mesoderm (brightfield) under increasing applied strain.

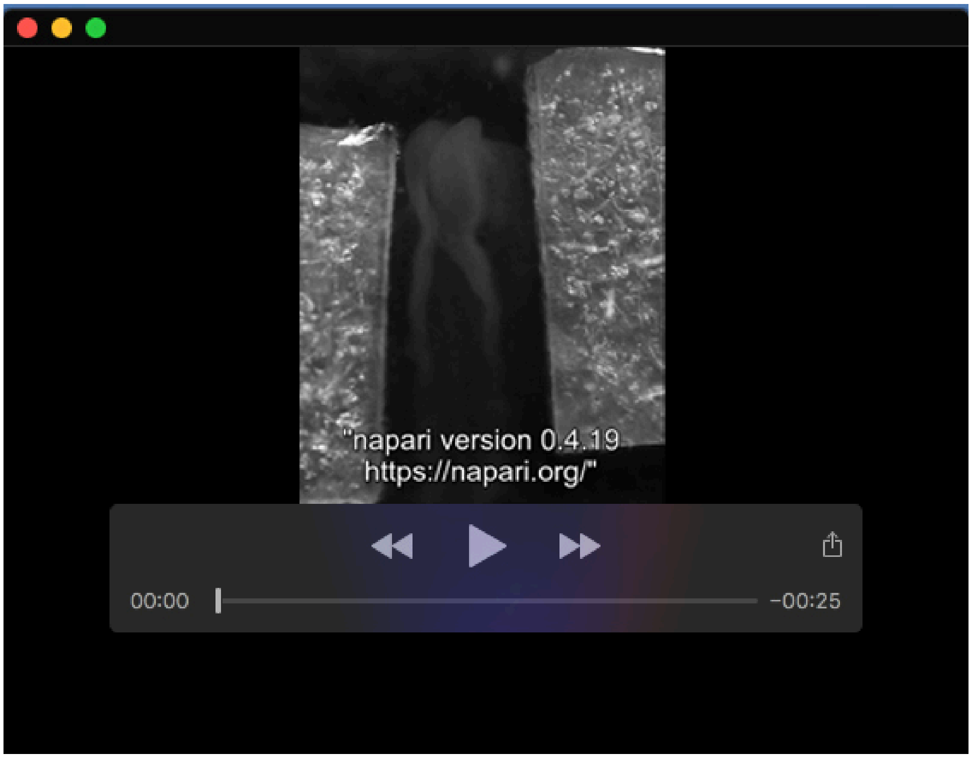

Movie 4. Time-lapse movie of unzipping the neural tube (brightfield) under increasing applied strain.

Development • Supplementary information
